# Here Today, Gone Tomorrow? Changes in 4-Month-Olds' Physiologic and Behavioral Responses Do Not Indicate Memory for a Social Stressor

**DOI:** 10.3389/fpsyg.2018.00128

**Published:** 2018-02-20

**Authors:** Jennifer A. DiCorcia, Nancy C. Snidman, Ed Tronick

**Affiliations:** Department of Psychology, University of Massachusetts, Boston, MA, United States

**Keywords:** infant memory, social stress, salivary cortisol, infant behavior, double Face-to-Face Still-Face (FFSF) paradigm

## Abstract

Although much is known about early memory development, only a few studies have explored infants' memory of social stress. While these few studies suggest that infants can remember stressful interactions, limitations seen in both methodology and statistical analyses give pause. In the current study, 4-month-olds and their mothers participated in both stressful and non-stressful interactions over 2 days. On Day 1, memory group infants participated in the double Face-to-Face Still-Face (FFSF) paradigm and control group infants participated in typical play. Both groups experienced the double FFSF paradigm on Day 2. Memory group infants exhibited the standard SF response but no differences in infant cortisol on Day 1. Both infant groups exhibited the standard SF response on Day 2. However, infants in the memory group, who saw the FFSF paradigm for the second time, did not demonstrate changes in cortisol or behavior indicative of memory across the 2 days. There was also no relationship between changes in cortisol and behavior for both days. The findings question the use of salivary cortisol as a measure of social stress and suggest that, although 4-month-olds reacted to the Still-Face social stressor immediately, they did not remember the following day.

## Introduction

The age at which infants and young children remember events is a topic of considerable interest to developmental and cognitive psychology, as well as to brain development and psychobiology. A large and important literature has found an increase in memory capacity with age and repeated exposure (Rovee-Collier, 1999; Carver and Bauer, 2001; Giles and Rovee-Collier, 2011; Lukowski and Bauer, 2013). However, the majority of this research is limited to infants' memory of people, objects, and actions. For example, in the first half year of life infants' recognize familiar faces (Pascalis and de Schonen, 1994) and their memory for specific actions increases from a few days to a few weeks (Hartshorn et al., 1998; Rovee-Collier, 1999). Infants also show the beginnings of deferred imitation (Pascalis and de Schonen, 1994; Learmonth et al., 2004). Considerably less is known about infants' developing memory for socioemotional experiences, specifically social stress. With the growth of the social neurosciences and the recognition of the fundamental importance of social experience for development, a question emerges as to whether memory for social stress, is similar to, partially overlapping, or even distinctly different than, memory for objects, events, and actions. Surprisingly only a few studies have looked at infants' memory of social stressors, and their findings, while intriguing, do not form a consistent picture due to different ages, methods, and indices of memory (Haley et al., 2011; Montirosso et al., 2013). Building on our own work and the work of others, in this study 4-month-old infants' memory for the maternal Still-Face (SF), an established infant social stressor, is examined using both behavioral and adrenocortical measures of memory.

The Face-to-Face Still-Face (FFSF) paradigm has consistently been a particularly fruitful methodological tool for evaluating young infants' reactivity and regulatory capacities and their ability to cope with an interactive stressor, the maternal Still-Face (SF) (Tronick et al., 1978; Cole et al., 2004). The original FFSF paradigm consisted of three 2-min episodes, face-to-face play, the SF, and another face-to-face play often referred to as the reunion episode (Tronick et al., 1978). Behaviorally, infants typically respond to the SF with what has come to be called the signature “still-face effect” (Adamson and Frick, 2003), a signature decrease in positive affect and an increase in negative affect and gaze aversion (Stack and Muir, 1990; Toda and Fogel, 1993; Weinberg and Tronick, 1996). In studies that have used micro-analytic scoring systems, infants have shown additional signs of distress in response to the SF with an increase in visual scanning, pick-me-up gestures, distancing behavior such as twisting and turning in their seat, and autonomic stress indicators such as spitting up (Toda and Fogel, 1993; Weinberg and Tronick, 1996; Weinberg et al., 1999). A number of physiologic and neuroendocrine measures have also been used to measure infants' reactions during the FFSF paradigm. Ramsay and Lewis (2003) measured infant salivary cortisol and found that 6-month-old infants had a significant increase in salivary cortisol in response to the SF, though only about half of the infants showed a cortisol increase. In an attempt to increase infant stress, Haley and Stansbury (2003) modified the standard FFSF paradigm to include two SF episodes (i.e., play, SF, play, SF, play). This modified procedure did elicit a significant change in cortisol, albeit modest, following the SF stressors.

While there are an abundance of studies that have examined the FFSF paradigm's immediate effect on infant behavior and physiologic systems, to the authors' knowledge only two studies have used similar indices of memory to examine the paradigm's lasting effects (Haley et al., 2011; Montirosso et al., 2013). These two studies differ from one another in critical ways that add to our current understanding of infants' memory, but each is not without its own weakness. Haley et al. (2011) found an anticipatory stress response in 6-month-old's response to the double FFSF paradigm following a 24-h delay. In their study during the first lab visit infants were separated into two groups, one group that participated in the double FFSF paradigm and another group that engaged in typical play with their mothers. The inclusion of the control group is an important feature of the study as it controls for environmental stress from the testing experience. Both groups returned to the lab the next day to see if the FFSF group *anticipated* the same stressful experience. Although there were no behavioral differences indicative of memory, as expected, infants exposed to the SF had greater changes in salivary cortisol on the second day compared to the control group. While the comparison between the memory group and control group is an important comparison, the authors failed to discuss changes in cortisol across days. That is, were the infants who showed an increase in cortisol on the second day the same group of infants who also showed an increase on the first day? Overlooking the pattern of cortisol reactivity between days dismisses the importance of individual differences in reactivity. Recent reviews suggest great variability in infants' cortisol reactivity highlighting the importance of exploring individual differences in reactivity instead of relying on mean differences (Gunnar et al., 2009; Jansen et al., 2010; Tollenaar et al., 2010). Additionally, having all infants partake in the double FFSF paradigm on the second day (i.e., second exposure to the paradigm for the memory group and first exposure for the control group), would have strengthened the interpretation of the comparison between groups on the second day. Nonetheless, despite the limitations, the study is important for its demonstration of changes in salivary cortisol as a potential anticipatory measure of infant memory.

The methodological limitations seen in Haley et al. (2011) were partially addressed in another study that also explored 4-month-old infants' memory for the double FFSF paradigm. Montirosso et al. (2013) observed changes in infant cortisol and behavior in response to the double FFSF paradigm following a 2 week delay. Unlike the Haley study, infants' reactivity to the FFSF paradigm was again measured during the second visit. The repeated exposure to the FFSF paradigm for the memory group along with the inclusion of a control group that experienced the paradigm once allowed for both within and between participant comparisons. However, the control group only visited the lab once and any changes in infants' behavior and cortisol reactivity may have been due to the FFSF paradigm itself, to the novel experience of visiting the lab, or both. Like Haley and colleagues, Montirosso and colleagues found no differences in behavior between the two visits, but they did not find significant differences in cortisol reactivity between groups perhaps due to the longer memory delay (24 h compared to 2 weeks) or age differences (6-month-olds compared to 4-month-olds). As a next step, individual differences in cortisol reactivity were considered by dividing memory group infants into two cortisol reactivity groups, increasers and decreasers, based upon infants' greatest change in cortisol following their first exposure to the paradigm. Once again behavioral differences were not found between cortisol reactivity groups, but there were differences in cortisol reactivity during infants' second exposure to the paradigm. During their second exposure to the paradigm, increasers again showed an increase in cortisol but to a lesser extent while decreasers now had increased cortisol. These differences across exposures were interpreted as memory. However, this study too was not without limitations. First, the control group visited the lab once. Perhaps the change in cortisol for the subsample of the memory group was due in part to the novelty of the lab experience and not in response to the social stressor. Variability in cortisol reactivity might be because of individual differences within the infant. In fact, several recent reviews question the use of cortisol as a measure of social stress in infants since most studies lack overall significant changes in cortisol and instead rely on the creation of reactivity groups (Gunnar et al., 2009; Jansen et al., 2010). This is an important point to consider because any difference between post-cortisol and baseline—no matter how small—was used to create cortisol reactivity groups in the Montirosso study. Day-to-day variation and assay error were not accounted for which may have overinflated the stress responder group. The observed changes need to exceed error of measurement, at the very least, to create reactivity groups. Methodologic recommendations for salivary cortisol suggest that differences need to be greater than ±0.02 μg/dL and post-paradigm cortisol must be at least 10% different than baseline in order to differentiate between an actual significant change in response to the stressor and assay error (Granger et al., 2012).

Even with their limitations, the findings from these initial studies suggest a developing capacity to remember social stress. Building upon past research, in the current study infants and their mothers were randomly assigned to either an experimental memory group (Group_Memory_) or a control group (Group_Control_). Dyads assigned to Group_Memory_ experienced the double FFSF paradigm on Day 1 and again on Day 2. Group_Control_ dyads experienced five 2-min episodes of face-to-face play with their mothers on Day 1 and the double FFSF paradigm on Day 2. Memory for the social stressor was assessed using changes in infant cortisol and behavior. The current study adds to previous research in several important ways. First, the current study employed a balanced research design where infants in both groups were tested twice over 24 h. This design offered two key memory comparisons—a between group comparison within each day and a within group comparison across the 2 days. Group_Memory_ infants were exposed to the double FFSF paradigm twice which allows for a test of memory capacity and stability of responses. Group_Control_ infants were also tested twice and served as a control comparison in two important ways. Their Day 1 experience controlled for possible behavioral or physiological effects associated with visiting a research lab (e.g., visiting a new place, meeting new people, use of physiological sensors, etc.) and on Day 2 their first experience with the double FFSF paradigm served as a memory control. Finally, like Montirosso et al. (2013), individual differences in cortisol reactivity during infants' first visit to the lab were explored. However, in the current study error attributed to cortisol sampling was accounted for in the creation of *Stress Response* and *No Stress Response* groups.

Three main questions were asked of the data, (1) did infants show the established SF behavioral response and typical increase in cortisol during their first exposure to the double FFSF paradigm, (2) were there changes in behavior and cortisol indicative of memory for the paradigm following a 24 h delay, and (3) did individual differences in infants' cortisol reactivity during their first exposure to the paradigm relate to changes in behavior indicative of memory during their second exposure to the paradigm? Taken together, like previous studies, it was hypothesized that all infants would show behaviors and physiology indicative of stress in response to their first exposure to the double FFSF paradigm (decreased positive affect and increased negative affect and gaze aversion, increased cortisol) (Adamson and Frick, 2003) (Haley and Stansbury, 2003; Lewis and Ramsay, 2005). In response to their second exposure to the paradigm, Group_Memory_ infants were expected to show a change in physiology and behavior from their Day 1 initial reaction to the paradigm.

## Method

### Participants

Participating infant-mother dyads were recruited from the newborn nursery of a large Boston metropolitan area hospital that serves a diverse population. All infants were full-term, clinically normal at delivery and healthy at the time of their visit. Sixty-four infants (48% female) and their mothers participated in the study (Group_Memory_ *n* = 44, Group_Control_ *n* = 20). Fourteen dyads were excluded from the study entirely due to maternal SF violations (*n* = 5), missing maternal video for SF violations assessment (*n* = 1), excessive infant crying during baseline play (*n* = 1), missing infant baseline and/or all post-paradigm cortisol samples (*n* = 5), and infants taking medications that affect cortisol levels (*n* = 2) (Hibel et al., 2006; Granger et al., 2009) which resulted in a final sample of fifty (Group_Memory_ *n* = 33, Group_Control_ *n* = 17) 4-month-old infants (*M* = 111.86 days, *SD* = 2.515) and their mothers (see Table 1 for demographics). Families were provided with a small stipend and infant gift for their participation. The study was approved by the University's IRB and all mothers provided written informed consent.

**Table 1:** Maternal demographics.

	**Group**_**Memory**_		**Group**_**Control**_	
	***n***	**%**	***n***	**%**
**ETHNICITY**				
Hispanic/Latino	2	6	1	6
Non-hispanic	31	94	16	94
**RACE**				
Asian	3^#^	9	0	0
Black or African American	2	6	3^#^	18
White	33	85	13	76
Not Reported/Declined	0	0	1	6
**HIGHEST EDUCATION OBTAINED**				
High school	1	3	1	6
Some college	1	3	2	12
College degree	12	36	5	29
Graduate school	19	58	9	53

# *Two mothers in Group_Memory_ identified as Asian and White. One mother in Group_Control_ identified as Black or African American and White*.

### Procedure

Mothers and their 4-month-old infants visited the laboratory (Child Development Unit) twice over 2 consecutive days. To keep time of day consistent for cortisol samples both within and across participants, infants were tested at the same time of day (±1 h) during both visits and, regardless of group, all infants were tested in the morning/early afternoon hours (between 8:30 a.m. and 1 p.m.). Two research assistants conducted each testing session, a primary research assistant who interacted with the mother and infant and a secondary research assistant who was responsible for the behind-the-scenes technical equipment (e.g., cameras, timing the paradigm). The primary research assistant was the same person for both testing days. Dyads were randomly assigned to either an experimental memory FFSF group (Group_Memory_) or a control group (Group_Control_) on Day 1. Upon arrival, mothers and their infants were escorted to a waiting room where a research assistant explained the study and answered any questions. Before obtaining written consent, mothers were also reminded that they may terminate the procedure at any time for any reason. Approximately 20–30 min after arriving at the lab and following consenting procedures, two baseline saliva samples were collected from the infant, each ~8 min apart. Dyads were then escorted to the testing room by a research assistant. Infants were placed in a highchair that was situated ~46 cm directly in front of their mothers. To allow unrestricted maternal touch during play episodes the highchair did not have a tray. Following techniques developed for the FFSF procedure, two cameras were positioned (one mother, one infant) to record the testing session (Weinberg et al., 1999). Video recordings were synchronized using an integrated system and software packages [MindWare ACQ software (MindWare Technologies, Ltd., Westerville, OH) and Mangold VideoSyncPro (Mangold International GmbH (Ed.)]. Before leaving the testing room the research assistant fit mothers with a small earpiece connected to a walkie-talkie so that she could discretely communicate with mothers during the testing session.

All dyads, regardless of group or day, participated in five, 2-min face-to-face episodes. To start the testing session, the research assistant instructed mothers to briefly look at a sign located on the wall to their right and then turn back to their infant. The sign served as a reminder to mothers about the study procedure and served as an episode starting cue for later video coding. Mothers then moved a yellow smock that hung on their back frontwards to their chest (in view of their infant) signaling the start of the testing session. The yellow smock served as a memory cue for Day 2. All mothers then started with an initial baseline play episode (Ep1_Play_) in which the research assistant instructed them to play in an unstructured way with their infants as they typically would at home. While mothers were allowed to touch and talk to their infants during play sessions, they were not allowed to introduce any outside toys or props and were asked to refrain from picking their infant up during the testing session. Infants who cried continuously for more than 30 s during the first baseline play were excluded from the study. All five episodes ended with a short break (2–3 s) during which mothers were instructed to stop what they were doing and look toward a sign located on their right. RAs then provided instructions for mothers to begin the next episode. This break ensured that both groups experienced five distinct episodes. This general procedure was repeated for both Group_Memory_ and Group_Control_ dyads for a total of the five 2-min episodes on both Day 1 and Day 2.

#### Group_Memory_

Dyads randomly assigned to Group_Memory_ participated in the double FFSF paradigm both on Day 1 (first exposure) and Day 2 (second exposure). For both days, following Ep1_Play_, mothers were instructed to begin the first 2-min SF episode (Ep2_SF1_). Mothers were asked to sit back in their chair and to maintain a neutral expression or “poker-face” while looking at their infant. Mothers were asked to refrain from talking to and/or touching their infant. To ensure fidelity, video recordings of mothers were reviewed for violation of the SF instructions. Mothers who violated the SF procedure were excluded from the study (e.g., changed from a neutral expression, touched, or otherwise interacted with their infant for more than a total of 20 s). Ep2_SF1_ was followed by another 2-min face-to-face play episode (Ep3_Play_), a second SF episode (Ep4_SF2_), and a final play episode (Ep5_Play_).

#### Group_Control_

Dyads randomly assigned to Group_Control_ participated in a total of five, 2-min face-to-face play episodes (Ep1_Play_ to Ep5_Play_) during their Day 1 visit. Dyads participated in the double FFSF paradigm during their Day 2 visit.

Group_Control_ served as a control in two different ways. First as a control for possible behavioral or physiological effects associated with visiting a research lab (e.g., visiting a new place, meeting new people, use of physiological sensors, etc.) but without experiencing the additional stress of the SF. Second, as a memory control in order to compare dyads experiencing the double FFSF paradigm either on just 1 day or on both days. Compared to past research that lacked a similar control group (Haley et al., 2011; Montirosso et al., 2013) the addition of the all play control group allowed for several unique comparisons. On Day 1 Group_Memory_ dyads experienced the stress of the SF and the stress of the lab visit whereas Group_Control_ only experienced the stress of the lab visit. Thus, the comparison of Group_Memory_ infants' reactivity on Day 1 to Group_Control_ infants' reactivity speaks to the effect of the SF in addition to the stress of the visit. On Day 2 both groups experienced the stress of both the SF and the lab visit, but Group_Memory_ infants experienced the SF for the second time while Group_Control_ infants did not. This provided two ways of exploring memory for the SF, first by comparing Day 2 performance across groups and by comparing the performance of Group_Memory_ infants across both days.

### Coding

#### Behavioral coding

Infant and mother behavior was videotaped and later coded by coders who were blind to study hypotheses and group membership. Gaze Faze, a modified Infant and Caregiver Engagement Phases (ICEP) coding system (Weinberg and Tronick, 1999), was used in which affect and gaze behaviors were coded separately. Gaze (toward mother for infant, toward infant for mother) along with positive (e.g., smiling, raised cheeks, and crinkly-eyes), negative (e.g., fussing, crying, frowning), and neutral affect were continuously coded using 1-s time intervals for both mother and infant using Mangold Interact software [Mangold International, GmbH (Ed)]. Mothers' and infants' faces must be visible for the majority of the episode time. Coders overlapped on at least 25% of total video files to evaluate inter-rater reliability (infant affect percent agreement = 75%, Cohen's κ = 0.58, infant gaze percent agreement = 73%, κ = 0.62 infant gaze).

#### Cortisol

Using a pre/post-stress design, four saliva samples were collected from infants. Saliva samples were collected with Sorbettes (Salimetrics, State College, PA), specifically designed for infants and small children. Two baseline samples were taken 8 min apart before the start of the testing paradigm followed by two post-paradigm samples taken at 20 and 35 min following the middle of the paradigm (i.e., the middle of Ep3_Play_). Twenty and 35 min were chosen to reflect reactivity and recovery of cortisol, respectively (Ramsay and Lewis, 2003; Gunnar et al., 2009).

Saliva samples were immediately stored in a −20°C degree freezer before being moved to a −80°C degree freezer for longer-term storage until shipped overnight on dry-ice for assay at the Institute for Interdisciplinary Salivary Bioscience. On the day of assay samples were thawed and centrifuged (to remove mucins) and assayed in duplicate for salivary cortisol using a commercially available immunoassay specifically designed for use with saliva without modification to the manufacturer's (Salimetrics, Carlsbad, CA) recommended protocol. The test volume was 25 μL, the range of sensitivity was from 0.007 to 3.0 μg/dL, and intra- and inter-assay coefficients of variation were on average less than 10 and 15% respectively.

### Data preparation

Cortisol samples were excluded for many methodologic reasons including missing samples (due to extreme infant distress), insufficient sample volume, and assay interference. Samples were also excluded if infants were fed within 10 min of the sample time. While rinsing infants' mouths with water should reduce any potential effects of milk or formula, doing so in close proximity to the sample time may dilute cortisol concentrations, as per assay manufacturer's guidelines (Salimetrics, LLC). After individual cortisol samples were excluded, participants needed at least one remaining baseline and one of the two post-paradigm cortisol samples (either 20 or 35 min) to be included in the study. As a result, a final sample of 33 Group_Memory_ infants and 17 Group_Control_ infants were included in cortisol only analyses. In addition to individual cortisol samples, two dependent variables were used to measure infant cortisol. In the first approach, since the specific time course for cortisol reactivity and recovery continues to be contested, infants' average baseline cortisol and highest peak cortisol were used to calculate a peak cortisol reactivity difference (i.e., average baseline—greatest peak post-paradigm sample). The second approach generated a cortisol reactivity category for each infant where baseline and peak post-stress cortisol values were used to categorize infants. The difference between the greatest post-paradigm cortisol sample and the average baseline sample needed to be both positive and greater than 10% for an infant to be classified as having a *Stress Response* and being different enough from the intra-assay coefficient of variation for the sample (Granger et al., 2012). If the difference was negative and/or less than 10%, the infant was classified as *No Stress Response*. This categorization approach differed from those used in previous work that categorized any increase, regardless of its size, as a responder without accounting for potential sample error attributed to the assay (Montirosso et al., 2013).

For behavioral data, duration of time was changed to proportion of time to account for differences in total episode times (e.g., episodes that were shortened due to extreme infant distress). Testing sessions were stopped early for infants who continuously cried for more than 30 s at any time after Ep1_Play_ (Group_Memory_ *n* = 5, Group_Control_ *n* = 2). There was no relationship between study drop-outs and group membership (Group_Memory_ = 15%, Group_Control_ = 12%, *p* >0.99). As a more conservative approach to the data, these seven dyads were dropped from behavioral analyses due to missing episode data. As a result, a final sample of 28 Group_Memory_ infants and 15 Group_Control_ infants and were included in behavioral analyses.

All data were explored within groups for possible univariate (> ±3 *SD* from the mean) and multivariate (Mahalanobis Distance, *p* < 0.001) outliers in addition to violations of normality across all dependent variables. To maintain as many data points as possible, outliers were kept and winsorized to ±3 *SD*s from the mean. There were no differences in overall findings for analyses conducted with winsorized values or without outliers. Behavioral data was transformed using an arcsine transformation appropriate for proportion data (2^*^(arcsine(p^1/2^)) to correct for normality issues (Howell, 2002). Cortisol values were corrected for normality using the common log (log_10_). Transformed data were used in analyses while raw data were used for descriptive statistics presented in figures and tables for clarity.

## Design and analysis

Preliminary results revealed no significant gender main effects or interactions so gender was not considered as a factor. Infant cortisol and behavioral data were analyzed using a series of univariate and mixed factorial GLMs with Group (Group_Memory_, Group_Control_), Day (Day 1, Day 2), Sample (Baseline, Post-1, Post-2), and/or Episode (Ep1_Play_, Ep2_SF1/Play_, Ep3_Play_, Ep4_SF2/Play_, Ep5_Play_) as factors. Multivariate results were used to address sphericity issues. A priori comparisons were explored using Bonferroni corrections [adjusted alpha levels = 0.0125 (two-way interaction), 0.001 (three-way interaction)]. Changes in infant cortisol were also explored using *t*-Tests and cortisol categories were explored using non-parametric analyses. Several questions were asked of the data related to Group_Memory_ infants' first exposure to the double FFSF paradigm and their memory of the paradigm following a 24 h delay.

## Results

### Question 1. Did group_memory_ infants show the established SF response and typical increase in cortisol in response to their first exposure to the paradigm?

#### Infant behavior

As an initial step, Group_Memory_ infants' first encounter with the double FFSF paradigm was analyzed before exploring their memory for the social stressor. A significant Episode main effect was found for each behavioral variable (see Table 2). As shown in Table 3, infants' exhibited the SF effect. Negative affect increased in response to the SF episodes and then subsequently decreased during play episodes while positive affect and infants' gaze to their mothers showed the opposite pattern. Although not typically reported as part of the SF effect, infants' neutral affect generally decreased over time. Infants were also more negative during the second SF compared to the first SF (Ep2_SF1_ vs. Ep4_SF2_: *M*_*D*_ = −0.528*, SE*_*D*_ = 0.088*, p* < 0.001).

**Table 2:** Statistical findings for Group_Memory_ infants' behavior during their first exposure to the double FFSF paradigm.

	**Episode**			
**Dependent variable**	**Wilks '**λ	***F***_(df)_	***p***	η*_*p*_*^2^
Gaze to mother	0.123	46.230 (4, 26)	*p* < 0.001	η*_*p*_*^2^ = 0.877
Negative affect	0.308	14.611 (4, 26)	*p* < 0.001	η*_*p*_*^2^ = 0.692
Positive affect	0.246	19.945 (4, 26)	*p* < 0.001	η*_*p*_*^2^ = 0.754
Neutral affect	0.378	10.681 (4, 26)	*p* < 0.001	η*_*p*_*^2^ = 0.622

*Group_Memory_ infants demonstrated the established behavioral SF effect during their first encounter with the paradigm*.

**Table 3:** Mean differences and standard errors for behavioral variables following significant episode main effects.

	**Gaze to mom**	**Negative affect**	**Positive affect**	**Neutral affect**
Ep1_Play_ vs. Ep2_SF1_	0.453 (0.049)^***^	−0.144 (0.031)^***^	0.253 (0.039)^***^	−0.108 (0.051)^*^
Ep2_SF1_ vs. Ep3_Play_	−0.399 (0.055)^***^	0.066 (0.031)^**^	−0.212 (0.040)^***^	0.145 (0.042)^**^
Ep3_Play_ vs. Ep4_SF2_	0.520 (0.043)^***^	−0.305 (0.048)^***^	0.225 (0.043)^***^	0.080 (0.064)
Ep4_SF2_ vs. Ep5_Play_	−0.415 (0.059)^***^	0.112 (0.061)^#^	−0.127 (0.036)^**^	0.032 (0.051)

# *significant at p < 0.1*.

* *significant at p < 0.05*.

** *significant at p < 0.01*.

*** *significant at p < 0.001*.

#### Infant cortisol

Three analytic approaches were used to explore changes in cortisol following Group_Memory_ infants' initial response to the double FFSF paradigm on Day 1. First, there was no difference between Group_Memory_ infants' baseline cortisol and their greater post-paradigm cortisol, F_(1, 32)_ = 0.007, *p* = 0.933, η_*p*_^2^ < 0.001 (see Table 4 for descriptive statistics). As a second approach, a one-sample *t*-test was used to compare Group_Memory_ infants' peak cortisol reactivity difference to a 10% notable difference. Results once again suggested that infants' did not show a cortisol stress response, *t*_(32)_ = −1.837, *p* = 0.076, d = 0.320. A final approach explored whether Group_Memory_ infants were more likely to be categorized as having a *Stress Response* than not. Equal numbers of Group_Memory_ infants were categorized as having a *Stress Response* (52%) and as *No Stress Response* (48%), $X_{\left(1,\text{N}\;=\;33\right)}^{2}$ = 0.030, *p* = 0.862.

**Table 4:** Descriptive statistics for cortisol.

	**Average baseline**		**Greatest peak**		**Peak reactivity**	
	**Mean**	***SD***	**Mean**	***SD***	**Mean**	***SD***
**DAY 1**						
Group_Memory_ (*n* = 33)	0.394	0.320	0.427	0.418	0.033	0.271
Group_Control_ (*n* = 17)	0.257	0.191	0.305	0.262	0.048	0.305
**DAY 2**						
Group_Memory_ (*n* = 33)	0.444	0.413	0.493	0.508	0.050	0.334
Group_Control_ (*n* = 17)	0.404	0.844	0.570	1.278	0.166	0.480

*Raw cortisol values (μg/dL) are presented for clarity*.

### Question 2. Were there changes in behavior and/or cortisol indicative of memory following the 24 h delay?

#### Was there behavioral evidence of memory for the FFSF paradigm?

Two different comparisons were used to explore memory for the FFSF paradigm, (1) Group_Control_ infants' behavior compared to Group_Memory_ infants' behavior on Day 2, and (2) changes in Group_Memory_ infants' behavior from Day 1 to Day 2.

**Figure 1:**
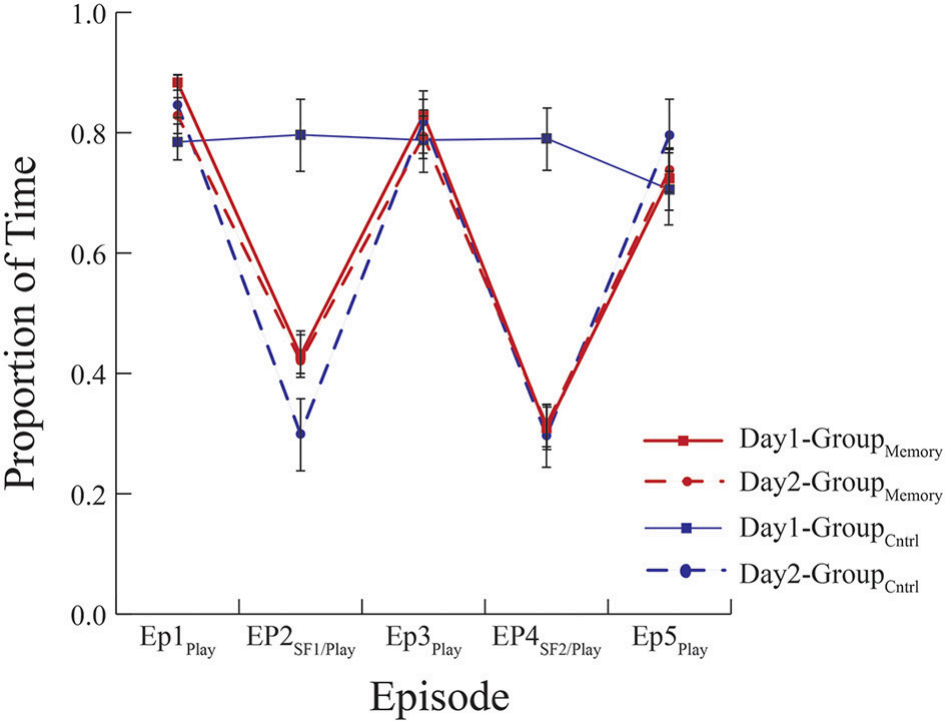
Mean proportion of time infants spent looking at their mother. Group_Memory_ infants spent less time looking at their mothers during the SF episodes on Day 1 and Day 2. Group_Control_ infants behaved similarly in response to their first exposure to the FFSF on Day 2. There were no differences in behavior indicative of memory between infants' first and second exposures to the FFSF paradigm.

**Figure 2:**
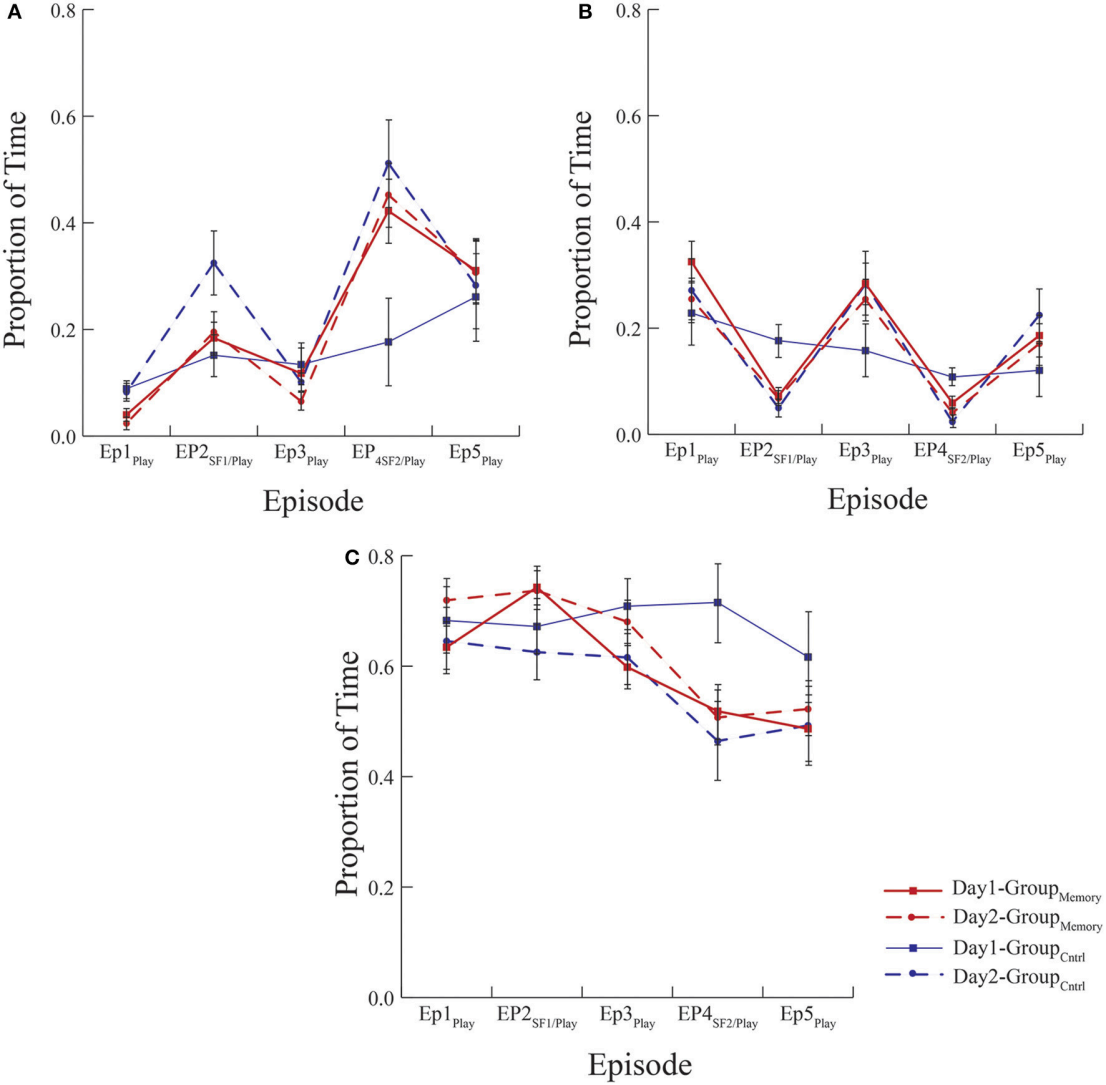
Mean proportion of time infants spent expressing **(A)** negative, **(B)** positive, and **(C)** neutral affect. All groups exposed to the FFSF paradigm showed the standard SF effect regardless of the number of exposures. There were no differences in behavior indicative of memory between Group_Memory_ infants' first and second exposure to the paradigm or Group_Memory_ infants' second exposure and Group_Control_ infants first exposure to the paradigm (see Day 2 comparisons between groups).

As a first approach, Group_Memory_ infants' behavior on Day 2 (i.e., their second exposure to the FFSF paradigm) was compared to Group_Control_ infants' behavior on Day 2 (i.e., their first exposure to the FFSF paradigm). Main effects and interactions are shown in Table 5. There were no differences in infant gaze (*ps* > 0.1) or infant positive affect (*ps* > 0.1) on Day 2 during any episode (see Figures 1, 2). Group_Control_ infants and Group_Memory_ infants looked at their mothers for the same amount of time and were equally positive on Day 2. For negative affect, Group_Control_ infants were more negative during Ep1_Play_ (*p* = 0.002), Ep2_SF1/Play_ (*p* = 0.084), and Ep3_Play_ (*p* = 0.060) than Group_Memory_ infants.

**Table 5:** Statistical findings for Group_Memory_ and Group_Control_ infants' behavior across episodes and days.

	**Episode x Group**			**Episode x Group x Day**		
	**Wilks' λ**	***F*_(1, 41)_**	$\eta_{partial}^{2}$	**Wilks' λ**	***F*_(4, 38)_**	$\eta_{Partial}^{2}$
Gaze to mother	0.836	8.065^**^	0.164	0.411	13.591^***^	0.589
Negative affect	0.920	3.569^#^	0.080	0.797	2.426^#^	0.203
Positive affect	0.996	0.177	0.004	0.655	5.014^**^	0.345
Neutral affect	0.855	6.979^*^	0.145	0.907	0.975	0.093

# *significant at < 0.1*.

* *significant at 0.05*.

** *significant at 0.01*.

*** *significant at 0.001*.

As a second approach, Group_Memory_ infants' behavior was compared across episodes and days (see Table 6). There was a main effect of Episode for all variables. Regardless of day, infants once again demonstrated the SF effect. There was also a significant Day x Episode interaction for positive affect where infants were more positive during Ep1_Play_ on Day 1 than Day 2 (M_D_ = 0.212, SE_D_ = 0.075, *p* = 0.009). Significant positive correlations between SF episodes across days were also found for most behavioral variables [Ep2_SF1_ Day 1 compared to Day 2: Gaze *r*_(28)_ = 0.313, *p* = 0.105, Negative Affect: *r*_(28)_ = 0.350, *p* = 0.068, Positive Affect: *r*_(28)_ = 0.449, *p* = 0.017, Neutral Affect: *r*_(28)_ = 0.282, *p* = 0.146; Ep4_SF2_ Day 1 compared to Day 2: Gaze *r*_(28)_ = 0.480, *p* = 0.010, Negative Affect: *r*_(28)_ = 0.338, *p* = 0.079, Positive Affect: *r*_(28)_ = 0.441, *p* = 0.019, Neutral Affect: *r*_(28)_ = 0.321, *p* = 0.096].

**Table 6:** Statistical findings for Group_Memory_ infants' behavior across episodes and days.

	**Day**			**Episode**		
	**Wilks' λ**	***F*_(1, 27)_**	$\eta_{partial}^{2}$	**Wilks' λ**	***F*_(4, 24)_**	$\eta_{partial}^{2}$
Gaze to mother	0.989	0.304	0.011	0.089	61.406^***^	0.911
Negative affect	0.992	0.219	0.008	0.215	21.960^***^	0.785
Positive affect	0.944	1.616	0.056	0.304	13.727^***^	0.696
Neutral affect	0.942	1.674	0.058	0.360	10.648^***^	0.640
	**Episode × Day**					
	**Wilks'** λ	***F***_(4, 24)_	$\eta_{Partial}^{2}$			
Gaze to mother	0.937	0.402	0.063			
Negative affect	0.853	1.034	0.147			
Positive affect	0.708	2.469^#^	0.292			
Neutral affect	0.835	1.184	0.165			

# *significant at < 0.1*.

*** *significant at 0.001*.

#### Was there physiological evidence of memory for the FFSF paradigm?

Like infant behavior, two different comparisons were used to explore possible physiologic indices of memory for the FFSF paradigm, (1) Group_Memory_ infants' Day 2 cortisol compared Group_Control_ infants' Day 2 cortisol and (2) Group_Memory_ infants' cortisol from Day 1 to Day 2. Three cortisol response variables were used, individual samples (average baseline, greater post-paradigm sample), peak cortisol reactivity difference (greatest post-paradigm peak—average baseline), and a cortisol reactivity category (*Stress Response, No Stress Response*). Descriptive statistics are shown in Table 4.

**Figure 3:**
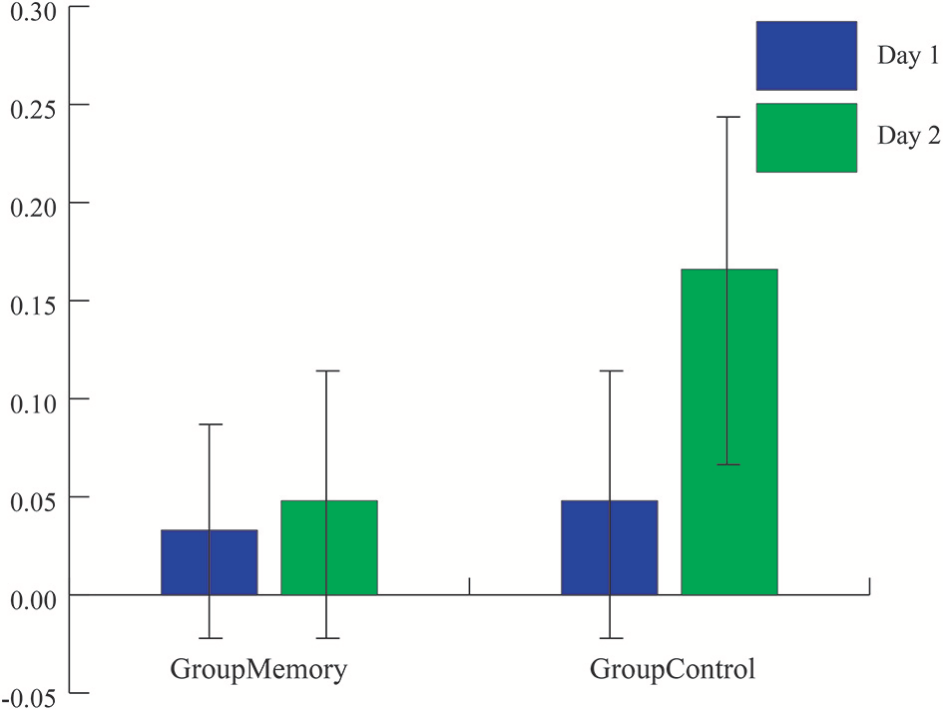
Average peak cortisol reactivity (baseline cortisol value-highest post-paradigm cortisol value) for each group across days. While the average peak cortisol value was higher for Group_Control_ infants on Day 2, there was not a significant group x day interaction (*p*s > 0.1).

**Figure 4:**
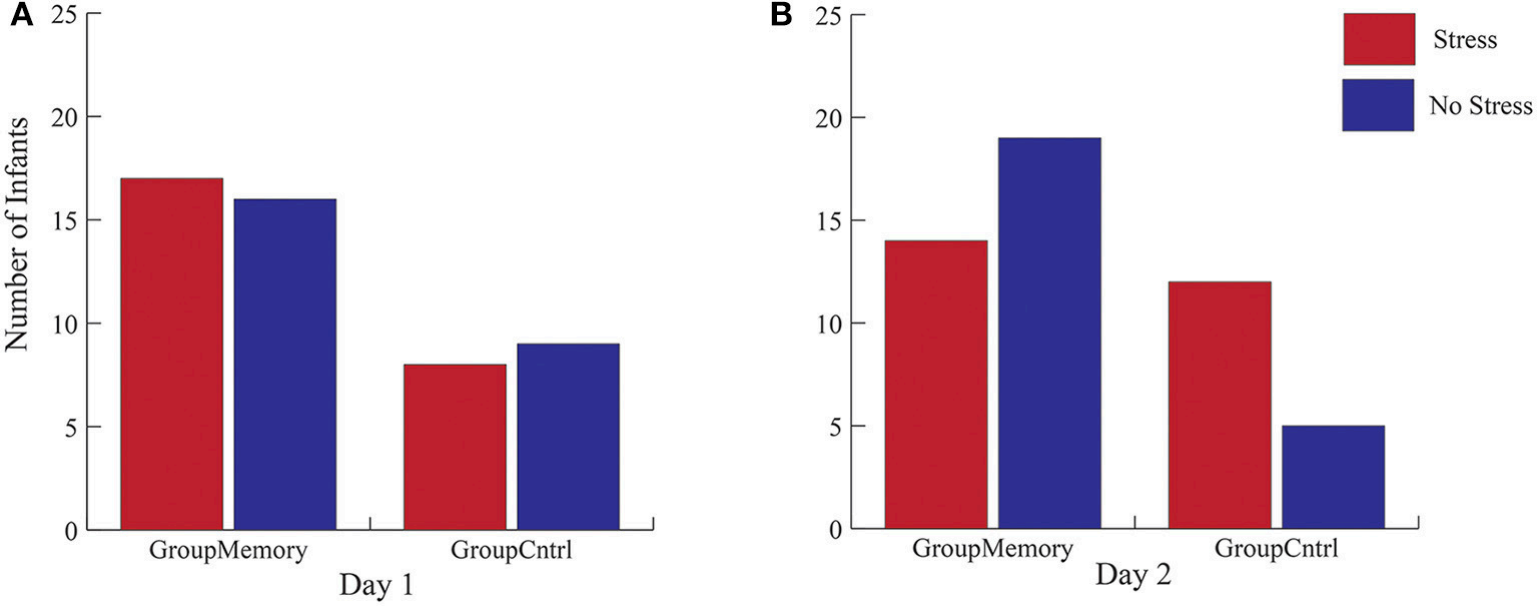
Cortisol stress categorization for both Group_Memory_ and Group_Control_ infants across days. **(A,B)** represent the Chi-Square results that compare cortisol categories across groups for each day (*N* = 50).

First, when comparing Group_Memory_ to Group_Control_ infants, the 2(Group) x 2(Day) x 2(Sample: Average BL, greater post-paradigm sample) interaction was not significant, Wilks' λ = 0.986, *F*_(1, 48)_ = 0.670, *p* = 0.417, η_*p*_^2^ = 0.014. Next, peak cortisol reactivity differences were compared across days and between groups. Once again, the 2(Group) x 2(Day) interaction was not significant, Wilks' λ = 0.984, *F*_(1, 48)_ = 0.793, *p* = 0.378, η_*p*_^2^ = 0.016. As shown in Figure 3, although infants' peak cortisol reactivity difference increased from Day 1 to Day 2 for both groups, the increase was not significant. Finally, chi-square tests of independence were used to compare infants' stress response category within groups for Day 2. As shown in Figures 4A,B, while there was no difference in stress response categories between groups for Day 1, $X_{\left(1,\text{N}=50\right)}^{2}$ = 0.089, *p* = 0.765, Φ = 0.042, there was a marginally significant finding for Day 2, $X_{\left(1,\text{N}=50\right)}^{2}$ = 3.566, *p* = 0.059, Φ = 0.267, where 71% of the Group_Control_ infants were categorized as having a cortisol *Stress Response* compared to 42% of Group_Memory_ infants.

When comparing Group_Memory_ infants' cortisol across days, the 2(Day) x 2(Sample) interaction was not significant, Wilks' λ = 0.990, *F*_(1, 32)_ = 0.335, *p* = 0.567, η_*p*_^2^ = 0.010. Peak cortisol reactivity differences were also compared across days and were not significantly different, *t*_(32)_ = −0.579, *p* = 0.567, d = 0.101. Finally, a McNemar test compared Group_Memory_ infants' stress categorization across days and once again found no difference, *p* = 0.549 (30% *Stress Response* both days, 36% *No Stress Response* both days, 21% *Stress Response* Day 1 only, and 12% *Stress Response* Day 2 only). Similar to behavior, significant positive correlations were found between samples and days (see Table 7). There was also a significant positive correlation between Group_Memory_ infants' peak cortisol reactivity difference across days, *r*_(33)_ = 0.395, *p* = 0.023.

**Table 7:** Group_Memory_ infants' baseline and greater post-paradigm cortisol correlations for Day 1 and Day 2.

		**Day 1**		**Day 2**	
		**Baseline**	**Greater post**	**Baseline**	**Greater post**
Day 1	Baseline	–	0.500^*^	0.508^*^	0.467^*^
	Greater post		–	0.325	0.641^**^
Day 2	Baseline			–	0.553^**^
	Greater post				–

* *significant at the 0.01 level*.

** *significant at the 0.001 level*.

### Question 3. Was there a relationship between infants' cortisol stress reactivity category on day 1 and their behavior on day 2 that would indicate memory for the FFSF paradigm?

The final set of questions explored relationships between Group_Memory_ infants' cortisol Stress Response Category on Day 1 (Stress Response, No Stress Response) and their behavior on Day 2. As shown in Table 8, there were no significant main effects or interactions. Stress response categorization on Day 1 had no effect on Day 2 behavior.

**Table 8:** Group_Memory_ statistical findings for main effects of Stress Response Category Day 1 and Episode Day 2 x Stress Response Category Day 1 interactions for all infant measures.

	**Stress response category Day 1**		**Stress response category Day 1** × **Episode Day 2**		
	***F*_(1, 26)_**	$\eta_{partial}^{2}$	**Wilks' λ**	***F*_(4, 23)_**	$\eta_{Partial}^{2}$
Gaze to mother	0.351	0.013	0.946	0.325	0.054
Negative affect	0.005	<0.0001	0.936	0.395	0.064
Positive affect	0.736	0.028	0.975	0.146	0.025
Neutral affect	0.306	0.012	0.902	0.623	0.098

## Discussion

The main goal of this study was to explore potential physiologic and behavioral indices of memory using an empirically validated social stress paradigm, the FFSF paradigm, in a sample of 4-month-old infants. Unexpectedly, and unlike previous research, the results of the current study suggest that infants did not remember the social stressor, the SF. There were no changes in cortisol reactivity when comparing Day 2 differences between Group_Memory_ infants and Group_Control_ infants. Adding to this, there were no differences in cortisol across days for Group_Memory_ infants. Instead, Group_Memory_ infants were consistent in their peak cortisol reactivity across the 2 days whether showing increases, no change, or decreases in cortisol from baseline.

Similar results were found using cortisol stress reactivity categories. More Group_Control_ infants were classified as having a cortisol stress response than Group_Memory_ infants. At face value, this finding might suggest that Group_Memory_ infants remembered the SF experience from Day 1 and were therefore less affected the second time around. However, once again, there was no change in Group_Memory_ infants' cortisol classification over the 2 days. On the contrary, the stability of infants' cortisol stress response seems to be a more likely explanation, perhaps due to temperamental differences in reactivity, rather than memory of the social stressor (Kagan and Snidman, 2004).

Stability of infants' responses is also a likely explanation for the behavioral findings. Like cortisol, there were no behavioral changes indicative of memory. When comparing Group_Memory_ infants' second exposure to Group_Control_ infants' first exposure to the FFSF paradigm, Group_Control_ infants were marginally more negative during the first three episodes. However, there were no differences in Group_Memory_ infants' behavior from their first exposure on Day 1 to their second exposure on Day 2. Additionally, when accounting for the relationship between cortisol stress reactivity and behavior across days, there was no difference in Day 2 behavior between infants categorized as having a cortisol stress response on Day 1 and those who were not categorized as having a stress response on Day 1.

The data from this study adds to the growing literature on the double FFSF paradigm as an empirically established social stressor as evidenced by behavioral measures. However, like some previous studies, infants' did not exhibit a cortisol response to their first exposure to the FFSF paradigm (Haley et al., 2011; Montirosso et al., 2013). There was also no relationship between infants' cortisol stress response categorization and behavior during their first exposure to the double FFSF paradigm. In terms of behavior, infants once again demonstrated the standard response to the SF (typical saw-toothed pattern for infant negative affect and the inverse pattern for positive affect and gaze to mother) regardless of the number of exposures to the FFSF paradigm. Infants were also more negative in response to the second SF episode compared to the first thus providing additional support for the double (A-B-A-B-A) FFSF producing more infant distress than the single (A-B-A) FFSF paradigm.

Methodologically speaking, this was one the few FFSF studies to include a control group that was exposed to the laboratory but not to the SF on their first visit. Although the control comparison was meant to be a pleasant experience for the dyad, surprisingly Group_Control_ and Group_Memory_ infants were equally negative on Day 1. However, as seen in Figure 2A, when considering the pattern of negative behavior, Group_Memory_ infants were more negative in response to the SF, especially the second SF, while Group_Control_ infants' negativity increased over time. This suggests that Group_Control_ infants were taxed and subsequently dysregulated by the repetitive face-to-face play whereas Group_Memory_ infants were reacting specifically to the SF. The control comparison also makes clear the potential stress brought about by the research experience. It is interesting to note that on Day 1 there was no difference in cortisol between groups (see Figure 3). Both findings suggest that infants were equally stressed on Day 1 as measured by cortisol. One possible interpretation of this finding is that the laboratory experience itself generated a stress response and experiencing the SF did not add to this stress.

Taken together, 4-month-old infants did not demonstrate physiologic or behavioral changes indicative of memory for the FFSF paradigm. Instead, infants' were more likely to demonstrate stability in their response to the social stressor. Why might this be the case? Perhaps the FFSF paradigm was not a strong enough stressor? This does not appear to be the case. Behavioral results suggest the opposite. Group_Memory_ infants demonstrated the classic SF response behaviorally on both days, but did not show an overall increase in cortisol in response to the paradigm. Adding to this, Group_Memory_ infants did not change their stress cortisol categorization across days. Therefore, a more likely explanation of the inconsistency in cortisol findings compared to previous studies points to the emerging or developing response of the adrenocortical system in response to social stress as seen in 4-month-old infants. Previous research using this same age group supports this interpretation (Montirosso et al., 2013).

The lack of memory noted in this study stands in stark contrast to the two previous studies. First, there were important methodological and statistical differences when comparing this study to the previous studies. Methodologically speaking, Montirosso et al. (2013) study created cortisol reactivity groups based on *any* difference between pre- and post-cortisol samples. This way of creating groups did not consider potential assay error. Instead, groups were created by using any difference between pre- and post-cortisol samples. The current study addressed these concerns in the creation of the *Stress Response* and *No Stress Response* cortisol groups (see, Granger et al., 2012). The failure to account for error in prior studies could have resulted in an inaccurate representation and interpretation of the cortisol data. Second, there were differences in control comparisons amongst studies. The control comparison group in Montirosso et al. study visited the lab only one time thus ignoring the potential stress brought about by the testing environment itself. The findings from the current study supported the idea of stress generated by the testing environment with almost half of the Group_Control_ infants classified as having a cortisol stress response during the all play episodes on Day 1. Haley et al. (2011) rectified this issue by having a control group visit the lab twice. However, neither the experimental FFSF group nor the control group experienced the FFSF paradigm the second day. Adding to this, there were no cross-day comparisons. Therefore, although Haley et al. found cortisol differences between groups on the second day, the paper lacked within comparisons to isolate variability due to individual differences. Simply put, there was no way of knowing if cortisol responses *changed* from Day 1 to Day 2. The current paper made both within and between participant and day comparisons to create a more thorough understanding of cortisol reactivity in response to the FFSF paradigm. By repeating visits and exposure to the paradigm, it was possible to tease apart potential effects of the lab and individual differences to better understand differences attributed to memory.

The current study is not without limitations. Though typical of many developmental studies, the sample size was small. The small number of participants most likely contributed to the variability seen in the data, however effect sizes were also small. In addition to finding a large amount of variability in cortisol values, there was also a lot of variability in behavioral responses. Infants became distressed at different points in the paradigm, if at all. Instead of picking an arbitrary point in time to represent the start of the cortisol reactivity clock, perhaps tethering infants' actual distress to the timing of their post-paradigm cortisol samples may reveal a clearer understanding of 4-month-olds' developing neuroendocrine response. The current study only explored one age group using a single paradigm to explore memory for social stress. Therefore, the findings must be interpreted with caution when extending to other age groups and forms of memory. Previous research suggests more consistent cortisol responses with older infants (Provenzi et al., 2016) and physical rather than social stress (Gunnar et al., 2009; Jansen et al., 2010) which could be the focus of future studies exploring memory in infancy. Along the same line, when considering memory for socio-emotional events, Hertenstein and Campos (2004) found that 14-month-olds, but not 11-month-olds, remembered an emotional message following a brief 1 h delay. DiCorcia and Mumme (reviewed in Mumme et al., 2007) also found that younger infants had a difficult time remembering negative messages. In their study, following an even shorter delay of 15-min, 12-month-olds' seemed to remember an actor's focus of attention during a negative reaction but not their emotional intent. Again, the findings from these studies point to a maturation effect in the development of memory for emotional events. Perhaps in the current study with even younger infants, these abilities have yet to come fully online.

In conclusion, while the current study failed to support previous research suggesting that 4-month-olds remembered the SF social stressor, it is important to try to make sense out of the lack of findings given the well-established, highly replicated, stressful behavioral and physiological effects of the FFSF paradigm. On face value, the paradigm should be a good candidate as a memorial event. Yet, here and elsewhere either no memory or only a weak memory effect was found. Contrast that with 4-month-olds' memory for people, events, and actions, what might accounts for the memorial differences? The number and amount of exposures to each might be a possible explanation. In this study, infants were exposed to the FFSF paradigm only once before the memory test. The literature on memory for objects, actions, and people suggests that number and duration of exposure has an effect on memory (Rovee-Collier, 1999; Haley et al., 2011). Certainly the people populating an infant's world are seen often and for longer durations. Perhaps repeated exposures and duration to the FFSF paradigm would have helped spur memory? The lack of findings for what is a well-established stressful event suggests that single exposures to emotional events are not sufficient to produce a long standing memorial effect. Moreover, as established as the stress of the FFSF is, not all infants react to it. There are striking individual differences. Adding to this, differences in cortisol reactivity have also been noted across early infancy (Jansen et al., 2010; Clements, 2013; Hill-Soderlund et al., 2014; Martinez-Torteya et al., 2015). Thus, one must be cautious in their attributions of permanent long- term effects of a singular stressful event because of both unknown developmental parameters that might affect the creation of long-term memories and individual differences in reactivity to the same event. Future studies should focus on these issues to gain a fuller understanding of the development of both the adrenocortical response and memory in infancy.

## Ethics statement

This study was carried out in accordance with the recommendations of the Institutional Review Board at the University of Massachusetts, Boston. All participants (mothers) gave written informed consent in accordance with the Declaration of Helsinki. The protocol was approved by the University of Massachusett's Institutional Review Board.

## Author contributions

All authors were involved in the design of the project and oversaw data collection. The corresponding author conceptualized and carried out data analyses and drafted the paper. All authors worked together toward the final manuscript.

### Conflict of interest statement

The authors declare that the research was conducted in the absence of any commercial or financial relationships that could be construed as a potential conflict of interest.
